# Influence of cyclin D1 splicing variants expression on breast cancer chemoresistance via CDK4/CyclinD1‐pRB‐E2F1 pathway

**DOI:** 10.1111/jcmm.17716

**Published:** 2023-03-13

**Authors:** Jing Wang, Jiaxin Zhang, Qinglong Ma, Shasha Zhang, Fengdie Ma, Wei Su, Taotao Zhang, Xiaodong Xie, Cuixia Di

**Affiliations:** ^1^ School of Basic Medical Sciences Lanzhou University Lanzhou China; ^2^ Bio‐Medical Research Center, Institute of Modern Physics Chinese Academy of Sciences Lanzhou China; ^3^ School of Biological and Pharmaceutical Engineering Lanzhou Jiaotong University Lanzhou China; ^4^ Key Laboratory of Heavy Ion Radiation Biology and Medicine of Chinese Academy of Sciences Lanzhou China

**Keywords:** alternative splicing, breast cancer, chemoresistance, Cyclin D1, Cyclin D1b, G870A polymorphism

## Abstract

Cyclin D1 (CCND1), a mediator of cell cycle control, has a G870A polymorphism which results in the formation of two splicing variants: full‐length CCND1 (CCND1a) and C‐terminally truncated CCND1 species (CCND1b). However, the role of CCND1a and CCND1b variants in cancer chemoresistance remains unknown. Therefore, this study aimed to explore the molecular mechanism of alternative splicing of CCND1 in breast cancer (BC) chemoresistance. To address the contribution of G870A polymorphism to the production of CCND1 variants in BC chemoresistance, we sequenced the G870A polymorphism and analysed the expressions of CCND1a and CCND1b in MCF‐7 and MCF‐7/ADM cells. In comparison with MCF‐7 cells, MCF‐7/ADM cells with the A allele could enhance alternative splicing with the increase of SC‐35, upregulate the ratio of CCND1b/a at both mRNA and protein levels, and activate the CDK4/CyclinD1‐pRB‐E2F1 pathway. Furthermore, CCND1b expression and the downstream signalling pathway were analysed through Western blotting and cell cycle in MCF‐7/ADM cells with knockdown of CCND1b. Knockdown of CCND1b downregulated the ratio of CCND1b/a, demoted cell proliferation, decelerated cell cycle progression, inhibited the CDK4/CyclinD1‐pRB‐E2F1 pathway and thereby decreased the chemoresistance of MCF‐7/ADM cells. Finally, *CCND1* G870A polymorphism, the alternative splicing of CCDN1 was detected through Sequenom Mass ARRAY platform, Sanger sequencing, semi‐quantitative RT‐PCR, Western blotting and immunohistochemistry in clinical BC specimens. The increase of the ratio of CCND1b/a caused by G870A polymorphism was involved in BC chemoresistance. Thus, these findings revealed that CCND1b/a ratio caused by the polymorphism is involved in BC chemoresistance via CDK4/CyclinD1‐pRB‐E2F1 pathway.

## 1 INTRODUCTION

Breast cancer (BC) is currently the most common malignant tumour worldwide.^1^ Up to 10% of BC patients have inherited (germline) DNA mutations, and ~90% of BC cases are caused by acquired (somatic) genetic and epigenetic alterations.^2^ Immunosuppressive therapy and androgen steroids can promote the development of BC.^3^ Human epidermal growth factor receptor 2 (HER2) is an established molecular prognostic marker, and it is often used to predict the response to endocrine therapy or targeted therapy in BC.^4^ Pathological and imaging examinations are helpful to diagnose patients with HER2‐positive BC.^5^ Studies have shown that BC can be subclassified based on genetic defects that reflect prognostic and predictive information to ensure that patients receive personalized treatment and improve treatment efficacy.^6^, ^7^ Adriamycin (ADM) and other anthracycline antibiotics were generally classified as first‐line drugs for BC chemotherapy.^8^, ^9^ However, chemoresistance to drugs has significantly limited its effectiveness. The underlying molecular mechanism is complex and related to multiple processes; the aberrant regulation of cell cycle is one of the factors.^10^, ^11^ Currently, the mechanism by which abnormal cell cycle regulation leads to chemoresistance remains elusive. Therefore, studying the molecular mechanism underlying the relationship between the cell cycle and drug resistance is essential to overcoming drug resistance.

Cyclin D1 (CCND1) is a mediator of cell cycle control that regulates the transition from G1 to S phase and contributes to cell cycle progression.^12^ The binding of CCND1 to cyclin‐dependent kinase 4 (CDK4) results in the phosphorylation of the retinoblastoma protein (pRB).^13^ This causes the release of E2F1 transcription factors, allowing the transcription of genes required for cell cycle G1 to S phase progression, enhancing the chemoresistance.^14^, ^15^, ^16^ The *CCND1* gene undergoes alternative splicing leading to the formation of two splicing variants: full‐length CCND1 (CCND1a) and C‐terminally truncated CCND1 species (referred to as CCND1b).^17^ CCND1a is the common variant containing five exons, while CCND1b derives from the retention of intron 4 and contains a premature termination.^18^ CCND1b is lacking the Thr‐286 phosphorylation site necessary for nuclear export. This structural difference of CCND1b renders it to localize in the nucleus through the cell cycle, which may increase its oncogenic potency.^19^ The G870A polymorphism at the splice donor site of the exon 4/intron 4 boundaries, which is thought to affect the production of CCND1a and CCND1b, was identified as a predictor for increased cancer risk.^18^ The *CCND1* G870A was reported to modulate CCND1b expression, which is also associated with poor clinical prognosis.^20^, ^21^ Furthermore, upregulation of CCND1b has been observed in several cancers including BC, thyroid cancer, glioma cancer, prostate cancer and non‐small cell lung cancer.^21^, ^22^, ^23^, ^24^, ^25^

To date, there is little evidence regarding the role of alternative splicing of CCND1 in BC chemoresistance. In addition, the influence of G870A polymorphism on the production of CCND1 variants in BC chemoresistance is not fully understood. In this study, we sequenced the G870A polymorphism and analysed the expressions of CCND1a and CCND1b in the ADM‐resistant MCF‐7 BC cells (MCF‐7/ADM) and MCF‐7 cells. Furthermore, CCND1b expression and the downstream signalling pathway were analysed through Western blotting and cell cycle in MCF‐7/ADM cells with knockdown of CCND1b. Finally, *CCND1* G870A polymorphism, the alternative splicing of CCDN1 was detected through Sequenom Mass ARRAY platform, Sanger sequencing, semi‐quantitative RT‐PCR, Western blotting and immunohistochemistry in BC clinical specimens. Here, our study provided the first evidence that CCND1b/a ratio caused by polymorphism is involved in BC chemoresistance via the CDK4/CyclinD1‐pRB‐E2F1 pathway. CCND1b/a ratio could be used as a predictive biomarker and potential target for the therapy of BC patients.

## 2 MATERIALS AND METHODS

### 2.1 Cells culture and cytotoxicity assay

MCF‐7 BC cells were obtained from the Lanzhou Institute of the Chinese Academy of Sciences. The ADM‐resistant MCF‐7 BC cells (MCF‐7/ADM) were generated from Fenghui Biotechnologies, Inc. by incubation in the gradually elevated level of ADM. ADM from Shenzhen Main Luck Pharmaceuticals Inc. was used in this study. Cells were cultured in Dulbecco's modified Eagle's medium (DMEM, Minghai Biochem) containing 10% foetal bovine serum (Minghai Biochem) at 37°C, 5% CO_2_ in an incubator. The medium for MCF7/ADM cells was supplemented with a low ADM concentration of 500 ng/mL. MCF7/ADM cells were cultured under ADM‐free circumstances in a medium without medicine for 2 weeks before the initiation of the study. The cell cytotoxicity assay was investigated using the CCK‐8 assay (Solarbio). Briefly, cells were seeded in 96‐well plates and incubated for 24 h. Then, different concentrations of ADM were supplemented into the medium and cultured in the incubator for 48 h to detect cytotoxicity. Three replicates were set in each group at each concentration. The absorbance was determined with a multifunction microplate reader (Tecan Infinite M200, Swiss) at 450 nm. The half‐maximal inhibitory concentration (IC_50_) was used to evaluate the relative drug resistance.

### 2.2 Cells transfection and cell cycle analysis

MCF‐7/ADM cells were transiently transfected by Lipofectamine 2000® (Invitrogen; Thermo Fisher Scientific, Inc.), according to the manufacturer's protocol. The siRNA sequences of CCND1b were 5′‐UCUUCCACUGCUCCUAGAAdTdT‐3′.^26^ A scrambled oligonucleotide sequence was used as a negative siRNA control. The siRNA oligonucleotide sequences were synthesized by Genepharma Co., Ltd. After transfection, the efficiency of transfection was tested using RT‐PCR and Western blotting. Cells were harvested for cell cycle analysis after transfection of 48 h. Subsequently, cells were fixed and stained with 50 μg/mL of propidium iodide (Solarbio). Cell cycle analysis was performed using NovoCyte (ACEA Biosciences), and the results were analysed using NovoExpress 1.5.0.

### 2.3 Study population

A total of 234 peripheral blood specimens were collected from patients who were admitted to the Lanzhou General Hospital of the Lanzhou Military Region between September 2013 and April 2020. In addition, we randomly obtained 18 snap‐frozen tissue specimens that were genotyped, including nine chemosensitive specimens with the GG genotype and nine chemoresistant specimens with the AA genotype. The association of two variants (CCND1a and CCND1b) with chemoresistance was analysed at the mRNA and protein levels by RNA and protein extraction from these 18 tissue specimens. We also collected 24 paraffin‐embedded BC tissue specimens, including 12 chemosensitive specimens with the GG genotype and 12 chemoresistant specimens with the AA genotype. All patients were confirmed by pathological examination and received chemotherapy for at least two courses. All chemotherapy regimens were based on anthracyclines. All specimens that had not received anthracycline treatment or received less than two courses of treatment were excluded. The effectiveness of chemotherapy can be evaluated clinically, such as tumour shrinking, which is an indicator of a good response.^27^, ^28^ The chemotherapy efficacy after anthracycline treatment was evaluated and scored according to the RECIST criteria.^29^, ^30^, ^31^, ^32^ According to RECIST, the therapy's response is classified as a complete response, partial response, stable disease or progressive disease, then classified into chemoresistant or chemosensitive groups.^33^, ^34^, ^35^ The patients were chemosensitivity if there is the loss of all tumour masses or pathological lymph nodes (complete response), and if there is a tumour that becomes at least 30% smaller than the longest diameter of the tumour (partial response). Patients were chemoresistance if there were tumour increases by at least 20% of the longest diameter (progressive disease) and where the tumour size reduction is not sufficient to generate a partial response, but tumour size does not increase and becomes a progressive disease (stable disease).^27^, ^29^, ^30^, ^36^ We classified the therapy's response by clinical examinations and CT scan or Doppler ultrasound according to the availability of the hospital and the type of patients' insurance. The ethics committee of Lanzhou University School of Basic Medicine approved the study.

### 2.4 DNA extraction and genotyping

Genomic DNA was extracted from the cells and 2 mL of ethylenediaminetetraacetic acid‐K (EDTA‐K)‐treated peripheral blood specimens (*N* = 234) using a genomic DNA extraction kit (Tiangen Genomic DNA Kit; Tiangen Biotech). All DNA specimens were frozen at −20°C until use. The genotyping of peripheral blood specimens was performed using the Sequenom Mass ARRAY platform (Beijing Bomiao Biotechnology Co., Ltd.). Approximately 10% of the specimens were further confirmed by Sanger sequencing (General Biosystems (Anhui) Co., Ltd.). Cells were genotyped by Sanger sequencing methods. The primers used are listed in Table S1.

### 2.5 RNA isolation and semi‐quantitative RT–PCR and quantitative RT–PCR

Total RNA was extracted from BC tissues (*N* = 18) and cell lines using TRIzol reagent (Invitrogen), according to the manufacturer's instructions. Then, total RNA was reverse‐transcribed using RT MasterMix (Cwbiotech). Gene‐specific primers for CCND1a and CCND1b were also designed (Table S1). We used cDNA synthesized by reverse transcription as the template for PCR amplification. The products of PCR were visualized on a 1% agarose gel and analysed by ImageJ software (National Institutes of Health). The PCR‐amplified products were verified via sequencing. Quantitative RT‐PCR was accomplished using an UltraSYBR Mixture (Cwbiotech). The target gene expression was quantified using the 2^−ΔΔCT^ method. Each sample was run in triplicate.

### 2.6 Western blotting and protein–protein docking

Proteins from snap‐frozen BC tissues (*N* = 18) and cell lines were extracted by lysing cells with a pre‐cooled radioimmunoprecipitation assay (Beyotime Biotechnology) and separated by sodium dodecyl sulphate‐polyacrylamide gel electrophoresis. After transmembrane, membranes were blocked with 5% skimmed milk for 1 h, incubated with primary antibodies overnight at 4°C. Antibodies against the following proteins were used: β‐actin (Cat No: 66009‐1‐Ig; Proteintech); CCND1 (Cat No: ab16663; Abcam); rabbit polyclonal antibody to CCND1b generated by GenScript using intron 4 CCND1b specific C‐terminal peptide sequence^37^; CDK4 (Cat No: 1026‐1‐AP; Proteintech); E2F1 (Cat No: 32‐1400; Thermo Fisher Scientific); Anti‐Rb (phospho S807) (Cat No: ab184796; Abcam). The membrane was subsequently incubated with HRP‐linked anti‐rabbit IgG antibodies (Cat No: bs‐0295G‐HRP; Bioss) for 60 min at room temperature. Antibody detection was performed using a chemiluminescence kit (NCM Biotech Co. Ltd.). ImageJ software (National Institutes of Health) was used for the quantitative analysis of proteins. Pymol‐2.3.0 and Swiss‐Model were performed to predict the structure of mutant proteins, which were introduced in Zdock for docking. Pymol‐2.3.0 was used for interaction mode analysis.

### 2.7 Immunofluorescence and immunohistochemistry

Formalin‐fixed paraffin‐embedded 4‐μm thick tumour sections were subjected to CCND1 immunostaining, according to the manufacturer's instructions. The antibodies (CCND1a, 1:200; CCND1b, 1:150) were incubated overnight. Negative controls used non‐specific rabbit Immunoglobulin G instead of the primary antibody, and a BC pathologist performed a blind evaluation of the immunostained sections. Slides stained with CCND1a and CCND1b antibodies were evaluated and blinded by an independent BC pathologist. Tumours with >50% CCND1a staining were considered CCND1a positive. Since CCND1b is not generally found in normal cells, tumours were considered positive for CCND1b if any cells showed staining in the nucleus.^20^, ^38^, ^39^ Cells were seeded on glass coverslips, fixed using either 3% paraformaldehyde and blocked for 1 h in 4% BSA/PBS followed by incubation in Anti‐SC35 (1:100, Abcam) for 1 h at room temperature. Cells were washed in PBS and incubated in a secondary FITC conjugated antibody (1:200) for 30 min at room temperature. Cells were washed with PBS and counterstained with Anti‐Lamin A (1:500, Abcam). The specimens were examined with a confocal laser microscope (LSM, Carl Zeiss AG).

### 2.8 Statistical analysis

Each assay was performed and calculated in triplicate (*N* = 3). Hardy–Weinberg equilibrium was evaluated in two groups of subjects to detect the genotype distribution of *CCND1* G870A polymorphism using online software (http://ihg.gsf.de/cgi‐bin/hw/hwa2.pl). The clinical data and genotyping results were analysed using SPSS 22.0 software (IBM Corp.), independent specimen *t*‐test, chi‐square test and Fisher's exact test (specimen size <5) were used. *p* < 0.05 was considered statistically significant. Data were presented as the Mean ± SD and analysed with GraphPad Prism 5.0. The differences between the two groups were analysed with Student's *t*‐test.

## 3 RESULTS

### 3.1 Resistance of MCF‐7/ADM cells to ADM

**FIGURE 1:**
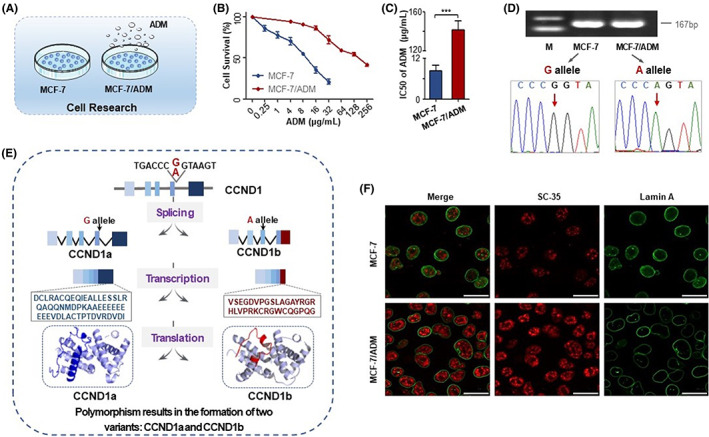
The G870A polymorphism affects CCND1 splicing variants in MCF‐7 and MCF‐7/ADM cells. (A) The schematic chart of MCF‐7 parental and induction of ADM‐resistant MCF‐7/ADM cells for cell research. (B) MCF‐7 and MCF‐7/ADM cells underwent treatment with ADM for 48 h and analysed by Cell Counting kit‐8 assay. (C) Half maximal inhibitory concentration values of MCF‐7 and MCF‐7/ADM cells for ADM are presented. (D) Analysis of the genotype of *CCND1* G870A polymorphism in MCF‐7 and MCF‐7/ADM cells by Sanger sequencing. (E) Schematic representation of the G870A polymorphism results in the formation of two variants: CCND1a and CCND1b. Different regions were labelled with CCND1a (blue) and CCND1b (red) in protein structure. (F) Immunofluorescence analysis of the nuclear speckle marker SC‐35 (red) in MCF‐7 and MCF‐7/ADM cells. Nuclear membrane was counterstained with Lamin A (green). The staining was observed with a confocal laser microscope. The representative images with similar results are shown. Scale bars represent 25 μm. The experiment is repeated and calculated in triplicate (*N* = 3). The data are expressed as the mean ± SD. ***p < 0.001.

To study the mechanism of BC chemoresistance, the ADM‐resistant BC cell line was generated by processing the parental MCF‐7 cells with consecutive rounds of ADM treatment (Figure 1A). MCF‐7 and MCF‐7/ADM cells were treated with ADM at different concentrations for 48 h and cell viability was assessed using the Cell Counting Kit‐8 (CCK‐8) assay. After cells had been treated with ADM, the growth of cells slowed down in a concentration‐dependent manner with an IC_50_ (Figure 1B and Table S2). Compared with MCF‐7 cells, MCF‐7/ADM cells were accompanied by higher IC_50_ values (Figure 1C). The above results indicated that MCF‐7/ADM cells were resistant to ADM. MCF‐7/ADM cells can provide a more ideal cell model for subsequent study of chemoresistance mechanisms in BC.

### 3.2 *CCND1* G870A polymorphism in MCF‐7/ADM cells

To investigate whether chemoresistance could cause alteration in the *CCND1* G870A polymorphism, we extracted DNA from MCF‐7 and MCF‐7/ADM cells. Then, the extracted DNA was PCR amplified with specific primers of *CCND1* G870A polymorphism. The PCR amplification products of MCF‐7 and MCF‐7/ADM cells were shown in Figure 1D. Subsequently, the PCR products were subjected to Sanger sequencing. Sequence alignment analysis showed that MCF‐7 cells carry the wild G allele, while MCF‐7/ADM cells carry the mutant A allele.

### 3.3 *CCND1* G870A polymorphism results in the formation of CCND1a and CCND1b

CCND1a is preferentially produced when the G allele creates an optimal splice donor site. In contrast, the A allele is expected to hinder the splicing event, allowing intron 4 retention, and production of a C‐terminally truncated product of CCND1b. Compared with CCND1a, CCND1b is encoded by the first four exons, lacks the complete exon 5 sequence and retains part of the intron 4 sequence (VSEGDVPGSLAGAYRGRHLVPRKCRGWCQGPQG). Using Swiss‐Model software, we predicted the protein structure of CCND1b. It is apparent from the protein model that CCND1b has an anomaly of the a‐helix at the C‐terminus (Figure 1E). The CCND1b protein has a completely divergent C‐terminal domain, lacking the PEST motif and residues (Thr‐286) that control nuclear export and protein stability. Therefore, CCND1b is considered to be a more stable constitutive nuclear protein with enhanced capability to regulate CDK activity and cell cycle control.

### 3.4 Upregulation of CCND1b/a ratio is associated with drug resistance in BC cells

**FIGURE 2:**
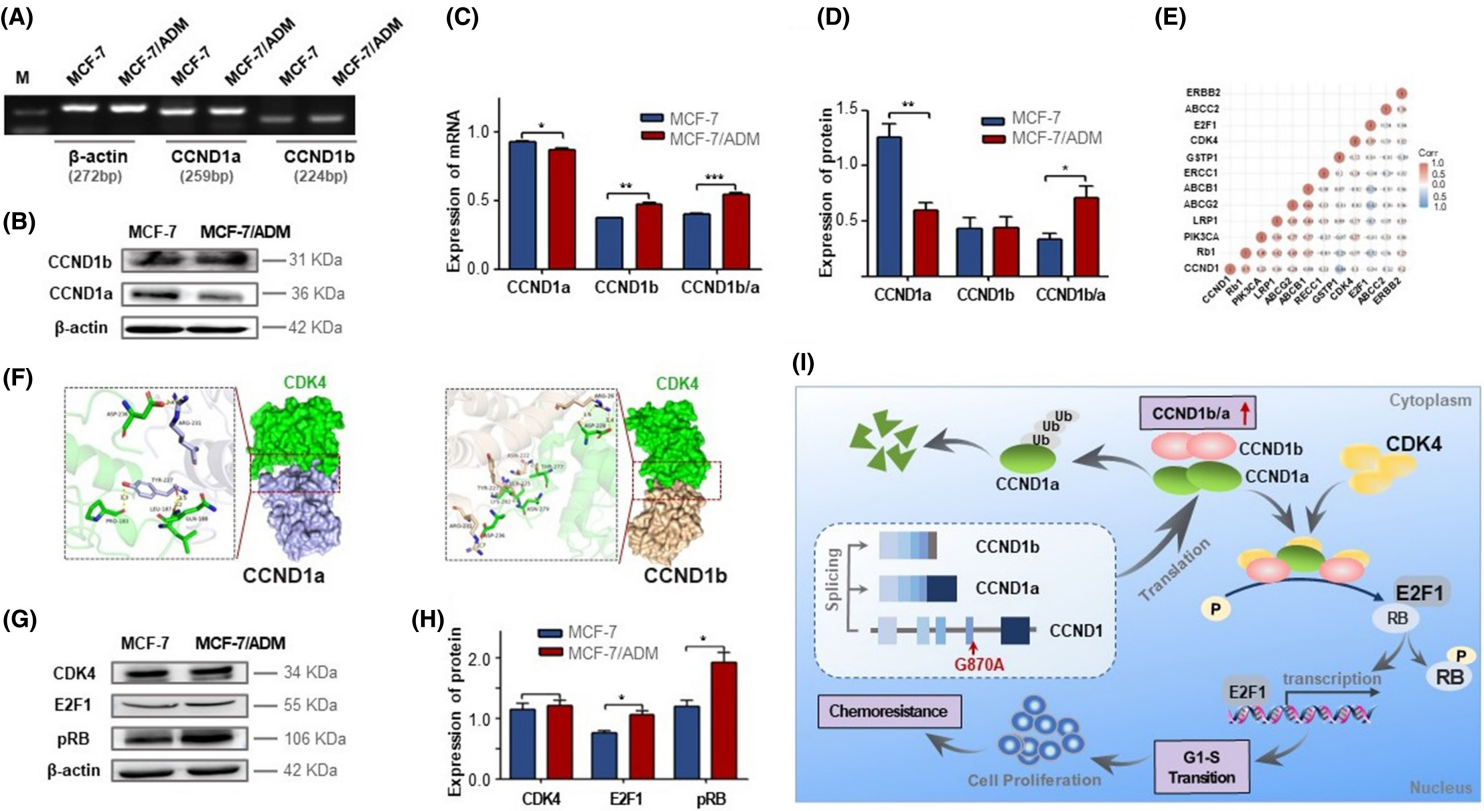
Upregulation of CCND1b/a ratio is associated with chemoresistance via cyclin D/CDK4‐pRB pathway in BC cells. (A) mRNA levels of CCND1a and CCND1b in MCF‐7 and MCF‐7/ADM cells were analysed by semi‐quantitative RT–PCR. (B) The protein expression levels of CCND1a and CCND1b in MCF‐7 and MCF‐7/ADM cells were analysed by Western blotting. (C) Quantitative analysis of the mRNA expression of CCND1a, CCND1b and CCND1b/a ratio in MCF‐7 and MCF‐7/ADM cells. (D) Quantitative analysis of the protein expression of CCND1a, CCND1b and CCND1b/a ratio in MCF‐7 and MCF‐7/ADM cells. (E) The correlation of *CCND1* with some BC‐associated drug resistance genes was analysed by the Assistant for Clinical Information online platform. (F) Model of CDK4 binding to the CCND1a and CCND1b. (G) The protein expression levels of pRB, CDK4 and E2F1 in MCF‐7 and MCF‐7/ADM cells. (H) Quantitative analysis of the protein expression of pRB, CDK4 and E2F1 in MCF‐7 and MCF‐7/ADM cells. (I) Working model of the upregulation expression of CCND1b/a ratio involved in BC chemoresistance via cyclin D/CDK4‐pRB pathway. The experiment is repeated and calculated in triplicate (*N* = 3). The data are expressed as the mean ± SD. ***p < 0.001; **p < 0.01; *p < 0.05.

We examined the subcellular localization of nuclear speckle marker SC‐35 protein by fluorescent immunohistochemistry to observe whether abnormal alternative splicing was present in MCF‐7 and MCF‐7/ADM cells. We found that the nuclear speckles were more rounded and more numerous in MCF‐7/ADM cells (Figure 1F). This indicated that there are abnormalities in transcript levels and alternative splicing in MCF‐7/ADM, which may be related to the expression levels of CCND1a and CCND1b. To gain more insight into the expression levels of CCND1a and CCND1b, we analysed the mRNA and protein expression of CCND1a and CCND1b by semi‐quantitative RT–PCR and Western blotting in MCF‐7 and MCF‐7/ADM cells. Reduced mRNA and protein levels of CCND1a were observed in MCF‐7/ADM, but the mRNA level of CCND1b was increased in MCF‐7/ADM. However, no difference was observed in CCND1b protein expression between the two groups (Figure 2A,B). Interestingly, the ratio of CCND1b/a was significantly upregulated in MCF‐7/ADM cells at mRNA and protein levels. Figure 2C,D were the gray values of mRNA and protein.

### 3.5 CDK4/CyclinD1‐pRB‐E2F1 signalling pathway activated in MCF‐7/ADM cells

We analysed the correlation between *CCND1* and some BC‐related drug resistance genes by Assistant for Clinical Information online platform. We focused on the association of some key genes in the CDK4/CyclinD1‐pRB‐E2F1 signalling pathway with *CCND1*. The *RB1*, *CDK4* and *E2F1* genes were relatively strongly correlated with *CCND1* in BC (Figure 2E). Therefore, our subsequent experiments mainly explored the relationship between the CDK4/CyclinD1‐pRB‐E2F1 signalling pathway and chemoresistance in BC. The formation of the CCND1/CDK4 complex is a key point in the CDK4/CyclinD1‐pRB‐E2F1 signalling pathway. We used ZDOCK software to dock CCND1a and CCND1b with CDK4 and performed interaction mode analysis with Pymol2.3.0. It was shown that both CCND1a and CCND1b can interact with CDK4 through different binding sites (Figure 2F). Furthermore, we analysed the protein expression of p‐RB, CDK4 and E2F1 in cells. E2F1 and p‐RB were highly expressed in MCF‐7/ADM (Figure 2G,H). However, the expression of CDK4 protein was not significantly different between the two groups. Combined with the experimental results, we speculated that the *CCND1* G870A polymorphism affects the ratio of CCND1a and CCND1b. Abnormal degradation mechanism of CCND1b because CCND1b lacks the C‐terminal PEST domain and Thr‐286. However, CCND1a can be degraded normally. This ultimately leads to the upregulation of CCND1b/a ratio, activation of the CDK4/CyclinD1‐pRB‐E2F1 signalling pathway and resulting in chemoresistance (Figure 2I).

### 3.6 Downregulation of CCND1b could demote cell proliferation and decelerate the cell cycle progression in BC cells

**FIGURE 3:**
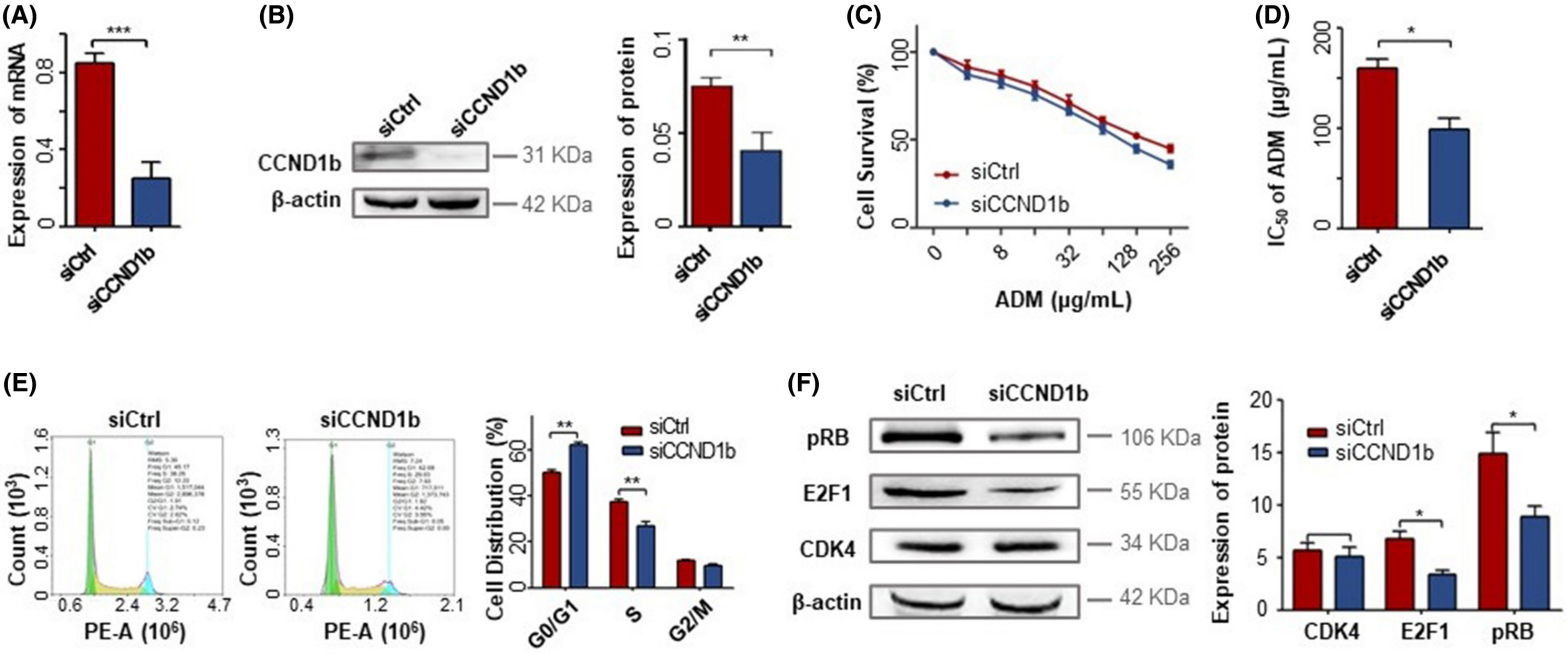
Downregulation of CCND1b could demote cell proliferation and decelerate the cell cycle progression in BC cells. (A, B) MCF‐7/ADM cells were transfected with siRNA targeting CCND1b, and the expression of CCND1b was analysed by RT–PCR and Western blotting. (C) MCF‐7/ADM cells of control and siCCND1b underwent treatment with ADM for 48 h and analysed cell proliferation by Cell Counting kit‐8 assay. (D) Half maximal inhibitory concentration values of MCF‐7/ADM cells of control and siCCND1b for ADM are presented. (E) Cell cycle progression was investigated by propidium iodide staining and flow cytometry in MCF‐7/ADM cells of control and siCCND1b. The percentages of cells at the different phases were calculated and plotted. (F) The expression of pRB, E2F1 and CDK4 proteins after knockdown of CCND1b. The experiment is repeated and calculated in triplicate (*N* = 3). The data are expressed as the mean ± SD. ***p < 0.001; **p < 0.01; *p < 0.05.

To further confirm the role of CCND1b in chemoresistance, we knocked down CCND1b in MCF‐7/ADM. Compared to control siRNA, CCND1b mRNA and protein levels were significantly reduced after 48 h of transfection with CCND1b‐specific siRNA (Figure 3A,B). To evaluate whether the inhibition of CCND1b enhances chemosensitivity, cells were transfected with CCND1b‐specific siRNA and analysed with CCK‐8 after treatment with ADM for 48 h. After cells had been treated with ADM, the growth of MCF‐7/ADM slowed down in a concentration‐dependent manner (Figure 3C). Compared with the control, the knockdown of CCND1b was accompanied by lower IC_50_ values (Figure 3D). This suggested that CCND1b plays an important role in BC chemoresistance and knockdown of CCND1b reverses ADM‐induced chemoresistance. Next, we measured the effect of CCND1b on cell cycle distribution. The results showed that the knockdown of CCND1b increased the number of cells in the G0/G1 phase, but decreased the number of cells in the S phase (Figure 3E). The aforementioned findings suggested that the knockdown of CCND1b could demote cell proliferation and decelerate the cell cycle progression, and reverse ADM‐induced chemoresistance.

### 3.7 Downregulation of CCND1b could inhibit cell‐cycle CDK4/CyclinD1‐pRB‐E2F1 pathway in BC cells

To demonstrate that CCND1b is essential for the activation of the CDK4/CyclinD1‐pRB‐E2F1 signalling pathway, we knocked down CCND1b in MCF‐7/ADM cells to observe the protein change of cell‐cycle CDK4/CyclinD1‐pRB‐E2F1signaling pathway. Knockdown of CCND1b resulted in decreased expression of E2F1 and pRB proteins, but no change in CDK4 protein (Figure 3F). Therefore, we assumed that the knockdown of CCND1b downregulated the ratio of CCND1b/a, inhibited the CDK4/CyclinD1‐pRB‐E2F1 signalling pathway, prevented tumour cells from passing through the G1‐S transition, resulting in cycle arrest, thereby decreasing the chemoresistance of MCF‐7/ADM cells.

### 3.8 *CCND1* G870A polymorphism is associated with chemoresistance in clinical BC specimens

**FIGURE 4:**
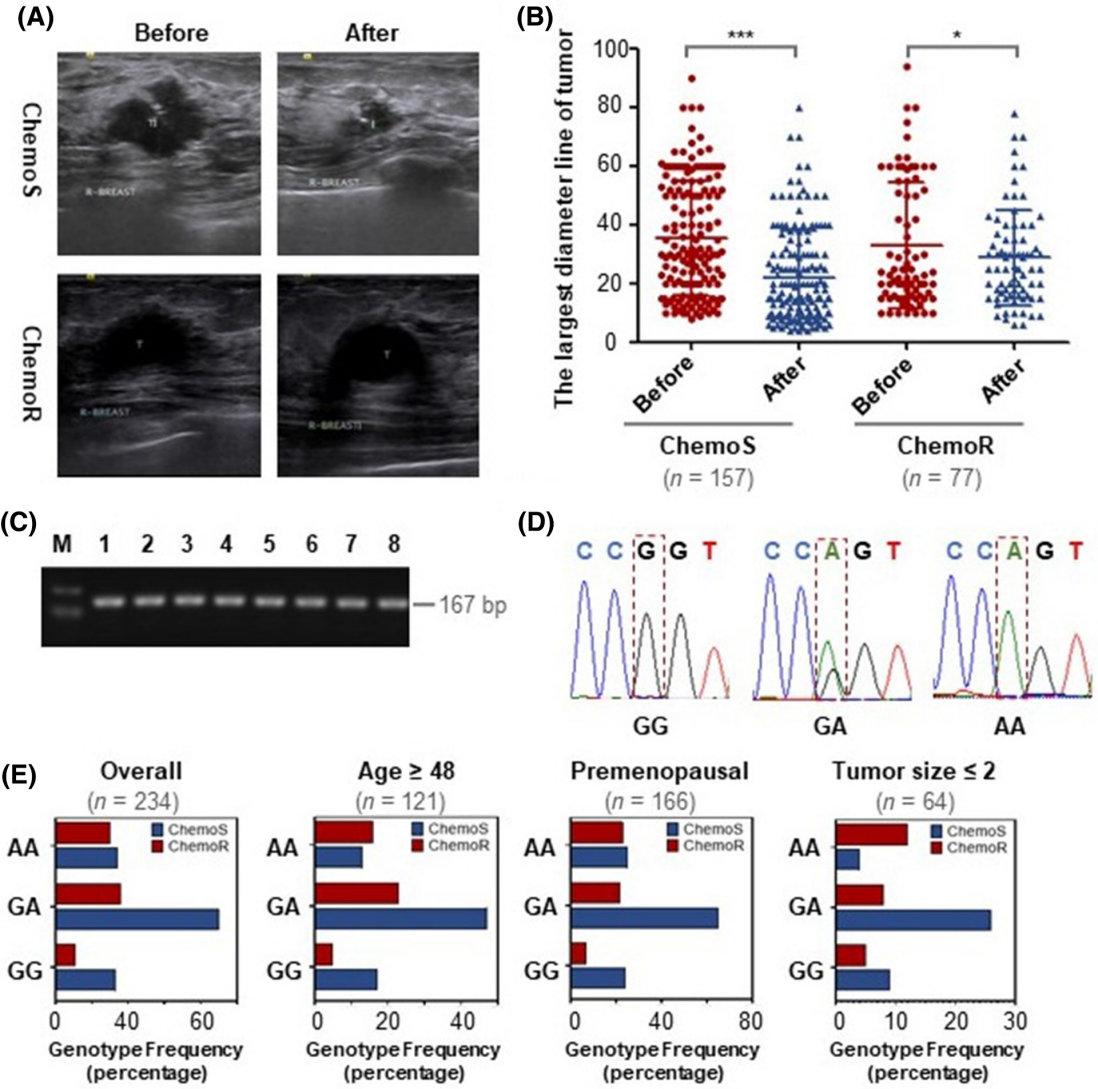
*CCND1* G870A polymorphism is associated with chemoresistance in clinical BC specimens. (A) Shown are representative tumour images harvested in BC specimens by the colour Doppler ultrasonography. The size of the tumour was estimated by measuring the largest diameter line of breast tumours, and the specimens were classified into chemosensitive and chemoresistant groups based on RECIST. (B) Statistical analysis of the largest diameter line of breast tumours in chemosensitive and chemoresistant specimens. (C) Electropherogram of PCR product of *CCND1* G870A polymorphisms in clinical BC specimens. (D) Sequence verification of *CCND1* G870A polymorphism: GG—wild homozygous, GA—mutation heterozygote, AA—mutation homozygous. The red box is the position of the G870A polymorphism base mutation. (E) The association between G870A polymorphism and BC chemoresistance risk based on subgroup analysis. The experiment is repeated and calculated in triplicate (*N* = 3). The data are expressed as the mean ± SD. ***p < 0.001; *p < 0.05.

The *CCND1* G870A polymorphism in all subjects was detected by the Sequenom Mass ARRAY platform, and the association between G870A polymorphism and chemoresistance in clinical BC specimens was analysed. The study included 234 patients with BC (157 chemosensitive and 77 chemoresistant patients), all of whom were Chinese. Table 1 shows the distribution of basic information on clinical BC specimens. The chemosensitive and chemoresistant groups were matched except for the histological type, PR and molecular subtype. The size of the tumour was estimated by measuring the largest diameter line of breast tumours according to the colour Doppler ultrasonography (Figure 4A). Figure 4B presents the distribution of the largest diameter line of breast tumours in chemosensitive and chemoresistant specimens. In chemosensitive specimens, the tumours shrank significantly after chemotherapy. However, the tumour shrinkage after chemotherapy was inapparent in chemoresistant specimens. First, we performed the test of Hardy–Weinberg equilibrium in our specimens. The genotypic frequencies of the chemosensitive group (*p* = 0.066) were in Hardy–Weinberg equilibrium, indicating that there was no sampling bias and no population stratification. The chemoresistant group was also in Hardy–Weinberg equilibrium (*p* = 0.970) (Table S3). Next, we analysed the correlation between the G870A polymorphism and BC chemoresistance in clinical BC specimens. The frequency distribution of different genotypes of *CCND1* G870A polymorphism is shown in Table 2. In the homozygote model, the AA genotype was significantly different from the GG genotype (OR = 2.647, 95% CI = 1.142–6.135, *p* = 0.021). The difference was statistically significant in the recessive and allele model (OR = 0.433, 95% CI = 0.239–0.785, *p* = 0.005; OR = 1.634, 95% CI = 1.102–2.423, *p* = 0.014). These results indicate that carrying the A allele or AA genotype can increase the risk of chemoresistance in BC. To further verify the accuracy of the genotyping method, we randomly selected some specimens to verify the *CCND1* G870A polymorphism. After PCR amplification of the specimen's DNA, the gel electrophoresis bands of the PCR products are shown in Figure 4C. The wild homozygous sequence, heterozygous sequence and mutant homozygous sequence of the *CCND1* G870A polymorphism are listed in Figure 4D.

**TABLE 1:** Characteristics of study subjects.

Characteristics	Total	Chemosensitive	Chemoresistant	*p*
Overall	234	157 (0.671)	77 (0.329)	
Age (years)				
<48	113 (0.483)	80 (0.510)	33 (0.429)	0.244
≥48	121 (0.517)	77 (0.490)	44 (0.571)	
Menopausal state				
Menopause	68 (0.291)	43 (0.274)	25 (0.325)	0.421
Premenopausal	166 (0.709)	114 (0.726)	52 (0.675)	
AJCC clinical stage				
II	104 (0.444)	64 (0.408)	40 (0.519)	0.395
IIIA	51 (0.218)	38 (0.242)	13 (0.169)	
IIIB	65 (0.278)	45 (0.287)	20 (0.260)	
IIIC + IV	14 (0.060)	10 (0.064)	4 (0.052)	
Histological type				
Invasive ductal carcinoma	222 (0.949)	153 (0.975)	69 (0.896)	**0.011**
Other types	12 (0.051)	4 (0.025)	8 (0.104)	
Tumour size				
T1 (size ≤ 2)	64 (0.274)	39 (0.248)	25 (0.325)	0.455
T2 (2 < size ≤ 5)	94 (0.402)	66 (0.420)	28 (0.364)	
T3 (size > 5)	76 (0.325)	52 (0.331)	24 (0.312)	
Lymph node staging				
N0	79 (0.338)	53 (0.338)	26 (0.338)	0.981
N1	81 (0.346)	55 (0.350)	26 (0.338)	
N2	58 (0.248)	39 (0.248)	19 (0.247)	
N3	16 (0.068)	10 (0.064)	6 (0.078)	
ER				
Negative	124 (0.530)	88 (0.561)	36 (0.468)	0.181
Positive	110 (0.470)	69 (0.439)	41 (0.532)	
PR				
Negative	70 (0.299)	55 (0.350)	15 (0.195)	**0.015**
Positive	164 (0.701)	102 (0.650)	62 (0.805)	
C‐erbB‐2^a^				
Negative	32 (0.137)	22 (0.140)	10 (0.130)	0.830
Positive	202 (0.863)	135 (0.860)	67 (0.870)	
Molecular subtype				
Luminal A	50 (0.214)	29 (0.185)	21 (0.273)	**0.041**
Luminal B	131 (0.560)	98 (0.624)	33 (0.429)	
HER2 overexpression	19 (0.081)	10 (0.064)	9 (0.117)	
Triple‐negative	34 (0.145)	20 (0.127)	14 (0.182)	

Abbreviations: C‐erbB‐2, human epidermal growth factor receptor 2; ER, oestrogen receptor; PR, progesterone receptor.

^a^ C‐erbB‐2‐positive: HER2 (+++) or Fish (+).

**TABLE 2:** The association between *CCND1* gene G870A polymorphism and BC chemoresistance.

SNP	Model	Allele/Genotype	Chemosensitive	Chemoresistant	OR (95% CI)	*p*
G870A	Heterozygote	GG	33 (0.210)	11 (0.143)	1.200 (0.548–2.629)	0.648
		GA	90 (0.573)	36 (0.468)		
	Homozygote	GG	33 (0.210)	11 (0.143)	2.647 (1.142–6.135)	**0.021**
		AA	34 (0.217)	30 (0.390)		
	Dominant	GG	33 (0.210)	11 (0.143)	1.597 (0.758–3.363)	0.215
		GA + AA	124 (0.790)	66 (0.858)		
	Recessive	AA	34 (0.217)	30 (0.390)	0.433 (0.239–0.785)	**0.005**
		GA + GG	123 (0.783)	47 (0.611)		
	Additive	GA	90 (0.573)	36 (0.468)	1.530 (0.884–2.647)	0.127
		GG + AA	67 (0.427)	41 (0.533)		
	Allele	G	156 (0.497)	58 (0.377)	1.634 (1.102–2.423)	**0.014**
		A	158 (0.503)	96 (0.623)		

Signifcant values are indicated in bold.

Subsequently, we examined the association between the G870A polymorphism and BC chemoresistance risk in the subgroups of the participants based on the relevant factors of age, menopausal status and tumour size. The stratification analysis by age, menopausal status and tumour size revealed that the distribution of the G870A genotype and allele between chemosensitive and chemoresistant specimens was slightly different (Figure 4E and Table S4).

### 3.9 Upregulation of CCND1b/a ratio affected by G870A polymorphism is associated with drug resistance in clinical BC specimens

**FIGURE 5:**
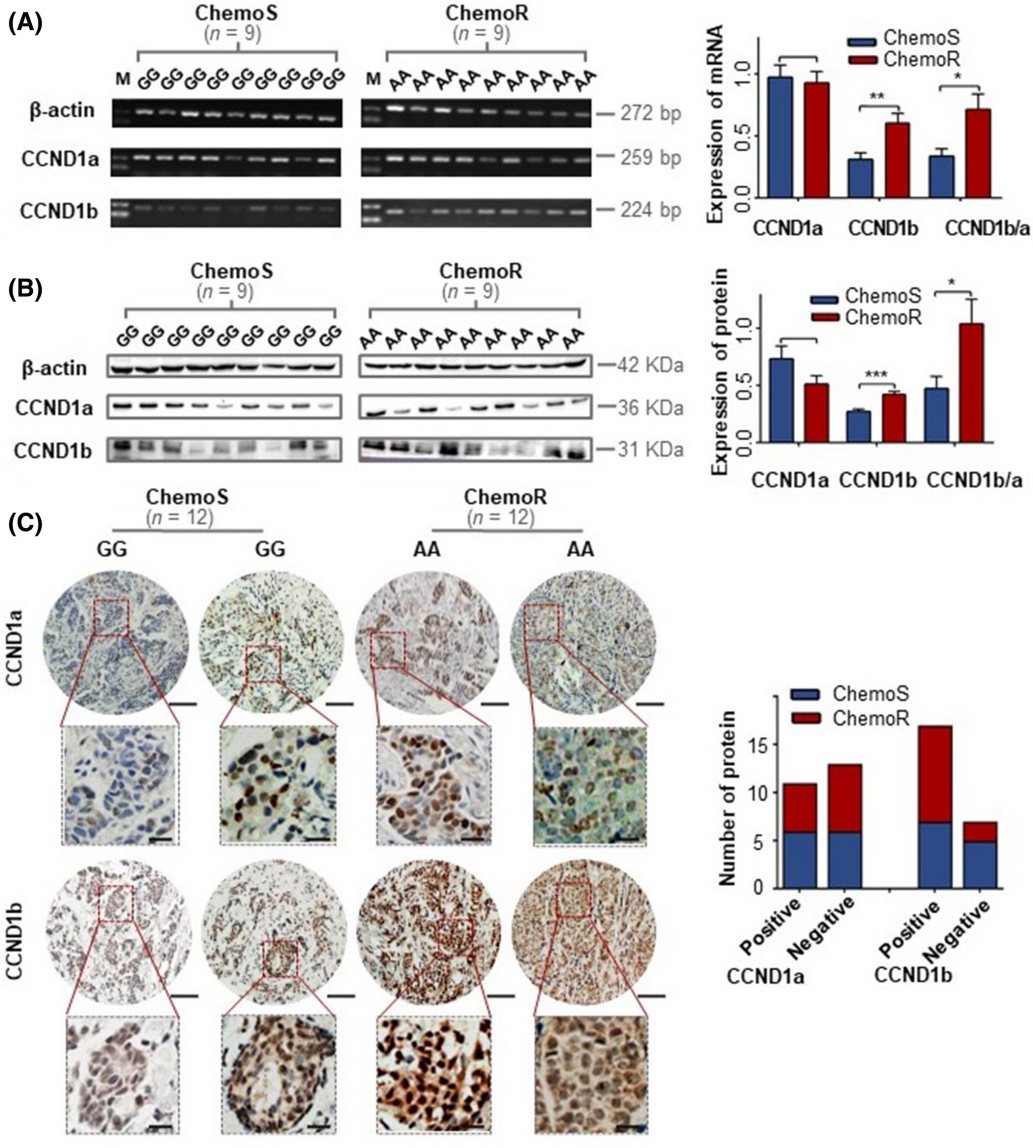
Upregulation of CCND1b/a ratio affected by G870A polymorphism is associated with chemoresistance in clinical BC specimens. (A) mRNA levels of CCND1a and CCND1b in chemosensitive (GG genotype) and chemoresistant (AA genotype) specimens were analysed by semi‐quantitative RT–PCR. The ratio of CCND1b/a was calculated. (B) Protein expression of CCND1a and CCND1b in chemosensitive (GG genotype) and chemoresistant (AA genotype) specimens was analysed by Western blotting. Calculated the ratio of CCND1b/a. (C) Immunohistochemical analysis of CCND1a and CCND1b. CCND1a was mainly distributed in the nucleus; CCND1b was presented in both the nucleus and cytoplasm. The representative images of immunohistochemical staining of CCND1a and CCND1b in BC specimens are shown. Scale bar represents 100 μm in the circle and 25 μm in the box. The experiment is repeated and calculated in triplicate (*N* = 3). The data are expressed as the mean ± SD. ***p < 0.001; **p < 0.01; *p < 0.05.

We randomly obtained 18 snap‐frozen tissue specimens that were genotyped to determine the expression of two variants (CCND1a and CCND1b), including nine chemosensitive specimens with the GG genotype and nine chemoresistant specimens with the AA genotype. The PCR products (CCND1a: 259 bp, CCND1b: 224 bp) were verified by sequencing. There was no alteration in the transcription level of CCND1a in the chemosensitive and chemoresistant specimens; however, there was a significant difference in CCND1b expression. The CCND1b/a ratio was higher in chemoresistant BC tissue specimens with the AA genotype (Figure 5A). Then, we detected the protein expression of CCND1a and CCND1b in 18 snap‐frozen tissue specimens using Western blotting. The result of proteins was consistent with the transcription level. CCND1b and the ratio of CCND1b/a were significantly increased in the chemoresistant specimens with the AA genotype (Figure 5B). Thus, we considered that the upregulation of CCND1b/a ratio affected by G870A polymorphism was associated with chemoresistance in BC. In the present study, we focused on the ratio of CCND1b/a, which is more likely to reflect the role of chemoresistance than CCND1b. To further explore the correlation of CCND1a and CCND1b with chemoresistance in BC patients, we stained chemosensitive (GG genotype) and chemoresistant (AA genotype) tissue specimens for CCND1a and CCND1b by immunohistochemistry. We found a high rate of positivity for CCND1b in chemoresistance specimens. In addition, CCND1a was mainly distributed in the nucleus, whereas CCND1b was distributed in the cytoplasm, cell membrane and nucleus. Figure 5C shows representative immunohistochemical staining for CCND1a and CCND1b. Due to our limited specimen size, no correlation between CCND1a and CCND1b expression and genotype was tentatively found.

## 4 DISCUSSION

CCND1, a nuclear protein that regulates cell cycle and controls the G1‐S transition, is closely related to the occurrence and development of various cancers.^40^, ^41^, ^42^ Up to 50% of BC is overexpressed with CCND1, and amplification of the *CCND1* gene is associated with prognosis.^42^, ^43^, ^44^ Studies have shown that the CCND1 expression was related to poor overall survival and disease‐free survival in BC.^45^, ^46^ In addition, the overexpression of CCND1 was associated with the response of chemotherapeutic agents such as adriamycin, 5‐fluorouracil and cisplatin.^47^ In recent years, an increasing number of studies have found that *CCND1* gene polymorphism was also associated with the occurrence and development of cancers, especially to chemotherapy response of cancers.^48^, ^49^, ^50^ However, the in‐depth mechanism between *CCND1* gene polymorphism and chemotherapy response remains to be elucidated. *CCND1* G870A polymorphism results in the formation of CCND1a and CCND1b,^51^ but the role of CCND1a and CCND1b variants in cancer chemoresistance remains unknown. We surmised that the CCND1 variants generated by the G870A polymorphism might be related to drug resistance. To date, there is little evidence regarding the role of CCND1a and CCND1b variants in BC chemoresistance. Therefore, this study aimed to explore the molecular mechanism of CCND1a and CCND1b variants in BC chemoresistance.

Previous studies reported that *CCND1* G870A polymorphism was associated with chemotherapy drug response in cancers.^48^, ^49^, ^50^ Absenger et al.^48^ found that *CCND1* G870A polymorphism can predict the clinical outcome of adjuvant 5‐FU chemotherapy in colon cancer patients. Garcia‐Aguilar et al.^49^ indicated that *CCND1* G870A polymorphism was associated with resistance to neoadjuvant chemoradiation therapy in rectal cancer. Labonte et al.^50^ showed that *CCND1* G870A polymorphism was useful in predicting clinical outcomes of lapatinib and capecitabine in HER2‐positive metastatic BC. However, the relationship between *CCND1* G870A polymorphism and ADM remains unclear in BC. ADM is a broad‐spectrum anti‐tumour drug widely used in chemotherapy for various malignant tumours.^52^ ADM and other anthracycline antibiotics were generally classified as first‐line chemotherapeutic drugs and were one of the most effective chemotherapeutic drugs in BC.^8^, ^9^, ^53^ Therefore, it is very important to explore the mechanism of ADM in BC treatment. In this study, we constructed a cell model and found a clear correlation between *CCND1* G870A polymorphism and ADM in BC. The wild G allele was carried in MCF‐7 cells, whereas the mutant A allele was carried in MCF‐7/ADM cells (Figure 1D). Then, we collected peripheral blood specimens to explore the relationship between *CCND1* G870A polymorphism and ADM in BC. Our study indicated that carrying the A allele or AA genotype can increase the risk of chemoresistance in clinical BC specimens (Table 2). Studies have shown that polymorphism can affect chemotherapy drug response, and there are significant differences among different populations.^54^ Therefore, it is necessary to explore the connection between *CCND1* G870A polymorphism and ADM in people with different genetic backgrounds. Our samples were mainly collected from patients in the north‐western region of China. It provides theoretical base for the establishment of *CCND1* gene polymorphism database in the northwest region of China and contributes to the pharmacogenomic research of personalized medicine.

*CCND1* G870A polymorphism results in the formation of CCND1a and CCND1b. The G870A polymorphism occurs at the boundary of intron 4/exon 5, which is located at the classical donor site and can generate two splice variants by the alternative splicing of mRNA.^18^ It is generally recognized that carrying the G allele creates an optimal splicing donor site, resulting in a CCND1 transcript containing all exons (CCND1a). When carrying the A allele enhances alternative gene splicing, to produce a shorter transcript (CCND1b) (Figure 1E).^17^ Comstock et al.^22^ cloned the intron 4 sequence containing the G or A allele and inserted it into the full length by minigene, and ultimately proved that the A allele preferentially produces CCND1b. In our study, we found that CCND1b expression can be increased by the A allele. However, the result is inconsistent and Howe et al.^51^ found that A allele produced more CCND1a while G allele had more CCND1b in malignant lymphocytes. This inconsistency may be caused by sample size and disease types. Therefore, it is necessary to analyse in a larger cohort of patients and diseases to eliminate the inconsistency of published literature.

CCND1a and CCND1b play an important role in cancer. CCND1a was discovered in 1991 and has high expression in various cancers.^55^ Since CCNDb was discovered in 1995, it has been widely studied in cancers.^17^ CCND1b is constitutively in the nucleus.^56^ In contrast to CCND1a, the CCND1b variant produces a protein lacking the C‐terminal PEST domain and the residue Thr‐286. Phosphorylation of Thr‐286 in the PEST domain enables the nuclear export of CCND1 and subsequent ubiquitin‐dependent degradation in the cytoplasm.^57^ CCND1b was thought a nuclear proto‐oncogene independent of CCND1a.^19^ Studies have shown that the CCND1b variant is more oncogenic or pro‐proliferative than CCND1a when transfected into cells, despite lower expression of CCND1b protein compared to CCND1a.^19^, ^56^ CCND1b is expressed in a variety of cancers, including B‐lymphoid malignancies, oesophageal cancer, mantle cell lymphoma cell lines, colon cancer, prostate cancer, cervical cancer, and breast cancer.^19^, ^37^, ^58^, ^59^, ^60^ At the same time, the CCND1b/a ratio is elevated in cancers.^26^ Moreover, abnormally high expression of CCND1b was particularly associated with poor outcomes in BC.^20^, ^21^ Studies showed that CCND1b antagonizes the action of CCND1a and inhibits the growth of breast cancer cells.^61^ However, the potential molecular mechanism of CCND1b and CCND1a in BC chemoresistance remains unclear. In our study, reduced mRNA and protein levels of CCND1a were observed in MCF‐7/ADM, but the mRNA level of CCND1b was increased in MCF‐7/ADM. However, no difference was observed in CCND1b protein expression between the two groups (Figure 2A,B). Interestingly, the ratio of CCND1b/a was significantly upregulated in MCF‐7/ADM cells at mRNA and protein levels. Our research indicated that there seems to be an equal amount of stable CCND1b in MCF‐7 and MCF‐7/ADM cells, which is due to the abnormal degradation caused by the deletion of PEST domain.^62^ The difference of CCND1a level may be related to the difference of G1 and G2 population size between MCF‐7 and MCF‐7A/DM cells. As we all know, CCND1 is a mediator of cell cycle control that regulates the transition from G1 to S phase and contributes to cell cycle progression.^12^ Studies had reported that the level of CCND1a was low in the S phase and high in G1 and G2 phases.^63^, ^64^ Therefore, the difference of G1 and G2 population size between MCF‐7 and MCF‐7/ADM may be one of the reasons for the difference of CCND1a level, which may have some impact on our conclusion. In this study, we only observed the difference of CCND1a levels through simple experiments. In the future, we will conduct in‐depth molecular mechanism research to determine the specific reasons for this difference. In cell research, we found that the ratio of CCND1b/a at the mRNA and protein levels is upregulated in MCF‐7/ADM cells (Figure 2A–D). Furthermore, we further performed validation in clinical specimens (Figure 5A,B). In clinical specimens, this result was consistent with the cell research results. Thus, this indicates that the upregulation expression of the ratio of CCND1b/a caused by G870A polymorphism was involved in BC chemoresistance.

The dysregulated cell cycle progression is associated with chemoresistance.^11^ The CDK4/CyclinD1‐pRB‐E2F1 signalling pathway axis is the key transcriptional mechanism that drives the process of cell cycle.^65^, ^66^ Interestingly, Lu et al. demonstrated that CCND1b retained the ability to combine to and activate CDK4. Different from CCND1a, CCND1b is constitutively localized in the nucleus throughout the cell cycle, and its constitutive expression promotes cell transformation.^19^ Moreover, a great deal of study has shown that although CCND1b was combined with CDK4, its role in inducing RB phosphorylation is limited.^19^, ^56^, ^67^, ^68^ In addition, Kim et al.^69^ indicated that CCND1b had lost its capacity to bind to CDK4, and CCND1b expression did not enhance the phosphorylation of RB protein in bladder cancer. However, the role of CCND1b in the CDK4/CyclinD1‐pRB‐E2F1 signalling pathway axis is still unclear. Based on crystal structures of Cyclin‐CDK complexes, the C‐terminus of CCND1 has not affected interactions with CDK subunits.^70^, ^71^ Therefore, CCND1b still retains the ability to bind and activate CDK4. Based on the predicted amino acid sequence, we used Pymol‐2.3.0 for homology modelling to predict the protein structure of CCND1b. The interaction of CCND1a and CCND1b with CDK4 was simulated using Zdock software. Protein interactions analysis showed that CCND1a and CCND1b can interact with CDK4 through different binding sites (Figure 2F). Amino acids PRO‐183, GLN‐188, LEU‐187, ASP‐236 of CDK4 form hydrogen bonds with TYR‐227 and ARG‐231 of CCND1a. Amino acids ASP‐236, LYS‐282, ASN‐279, THR‐277, ASP‐288 of CDK4 form hydrogen bonds with ARG‐231, TYR‐227, SER‐225, ASN‐222 and ARG‐26 of CCND1b. Amino acid of CDK4 and ILE‐295, ARG‐291, ARG‐228, PHE‐164, TYR‐226, LEU‐229, SER‐225, LEU‐224, PHE‐232, PRO‐169, PRO‐220 and ASN‐221 of CCND1a have a hydrophobic interaction, forming a hydrophobic surface. The above residues may constitute the active surface for the interaction between CDK4 and CCND1a. However, the amino acids ARG‐29, MET‐202, LEU‐32, SER‐201, TYR‐226, LEU‐224, PHE‐232, LYS‐167, PHE‐223, LEU‐229, MET‐168, PRO‐169, ASP‐25, LEU‐28, LEU‐22, TYR‐17, ASN‐21, ALA‐20 of CCND1b and CDK4 interact to form a hydrophobic surface. Although CCND1a and CCND1b can bind to CDK4 by model analysis, the difference of binding sites may lead to the difference of binding affinity. In this study, we observed that CCND1a and CCND1b had the same effect, both of which can promote cell cycle progression by binding to CDK4. When the CCND1b/a ratio was upregulated, CCND1b can phosphorylate RB by binding to CDK4, triggering the E2F1 required to enter the S phase to promote the cell cycle process,^37^ ultimately leading to drug resistance (Figure 2I). In addition, to confirm that CCND1b is essential for the activation of the CDK4/CyclinD1‐pRB‐E2F1 signalling pathway, we knocked down CCND1b in MCF‐7/ADM cells to observe the protein change of cell‐cycle CDK4/CyclinD1‐pRB‐E2F1signalling pathway. Our results showed that the knockdown of CCND1b downregulated the ratio of CCND1b/a, inhibited the CDK4/CyclinD1‐pRB‐E2F1 signalling pathway and prevented tumour cells from passing through the G1‐S transition resulted in cycle arrest, thereby decreasing the chemoresistance (Figure 3C–F).

## 5 CONCLUSION

**FIGURE 6:**
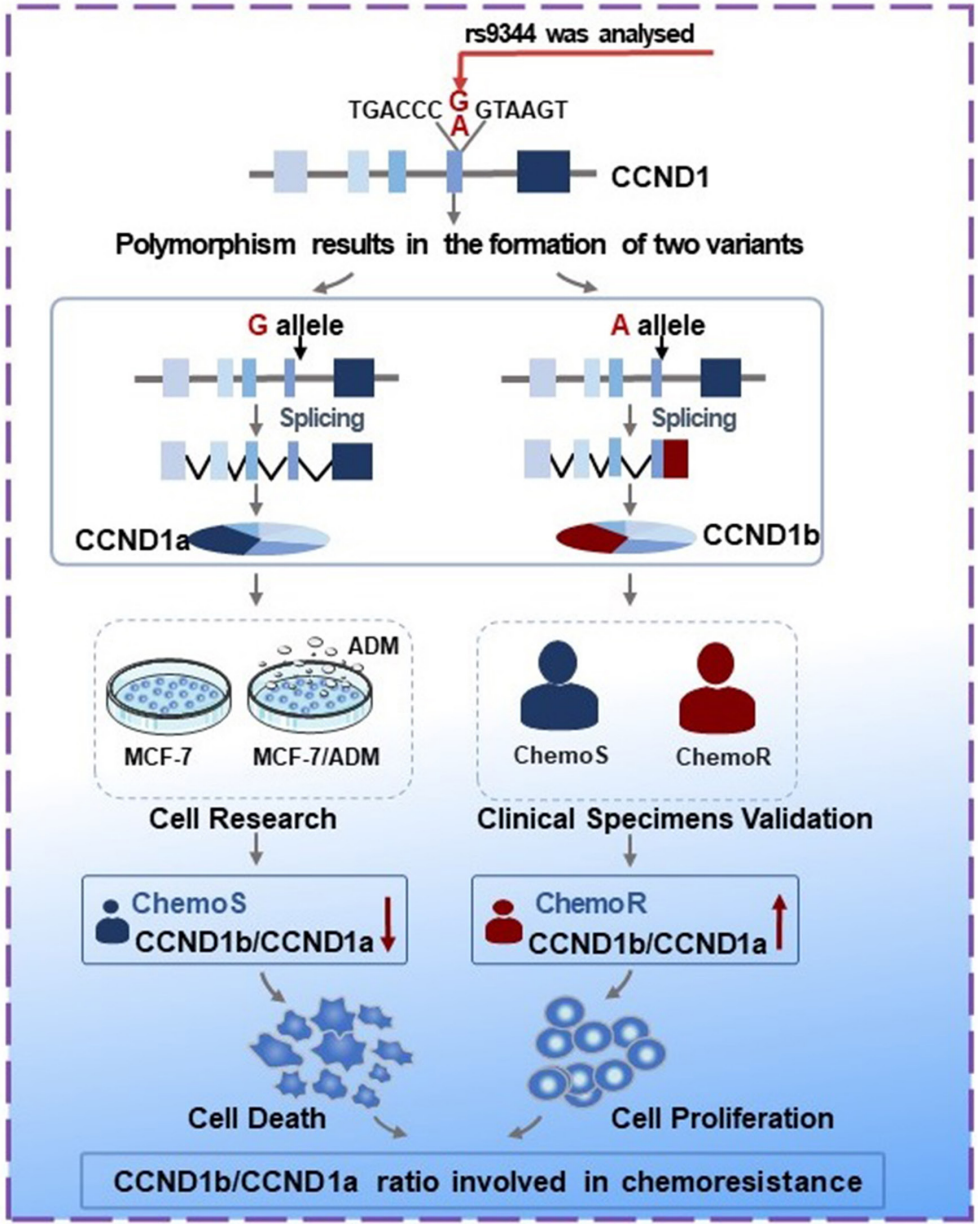
Schematic representation of the influence of CCND1 splicing variants expression on BC chemoresistance. Upregulation of CCND1b/a ratio affected by G870A polymorphism is associated with chemoresistance.

In conclusion, our study highlights CCND1b/a ratio was caused by G870A polymorphism involved in BC chemoresistance through cell research and clinical specimens' validation (Figure 6). Our data established CCND1b/a ratio as a potential predictive biomarker for chemoresistance and demonstrated that the CCND1b/a ratio may have a critical consequence for chemoresistance in BC. This study assessed the predictive value of CCND1 variants in BC chemoresistance and provided theoretical guidance for improving the efficacy of chemotherapy and achieving more precise individualized treatment in Northwest China.

## AUTHOR CONTRIBUTIONS

**Jing Wang:** Conceptualization (lead); data curation (lead); formal analysis (lead); writing – original draft (lead). **Jiaxin Zhang:** Data curation (supporting); investigation (supporting); methodology (supporting); supervision (supporting). **Qinglong Ma:** Data curation (supporting); investigation (supporting); methodology (supporting); supervision (supporting). **Shasha Zhang:** Data curation (supporting); investigation (supporting); methodology (supporting); supervision (supporting). **Fengdie Ma:** Investigation (supporting); project administration (supporting); supervision (supporting); validation (supporting); visualization (supporting); writing – review and editing (supporting). **Wei Su:** Investigation (supporting); project administration (supporting); supervision (supporting); validation (supporting); visualization (supporting); writing – review and editing (supporting). **Taotao Zhang:** Investigation (supporting); project administration (supporting); supervision (supporting); validation (supporting); visualization (supporting); writing – review and editing (supporting). **Xiaodong Xie:** Conceptualization (equal); funding acquisition (equal); project administration (equal); supervision (equal); writing – review and editing (lead). **Cuixia Di:** Conceptualization (equal); funding acquisition (equal); project administration (equal); supervision (equal); writing – review and editing (lead).

## FUNDING INFORMATION

This work was supported by the national Key R&D project of the Chinese Ministry of Science and Technology (2018YFE0205100), the Gansu Province Science and Technology Innovation Service Platform Construction Project (18JR2TA024), the Key Program of the National Natural Science Foundation of China (U1632270), the National Natural Science Foundation of China (11675234).

## CONFLICT OF INTEREST STATEMENT

The authors confirm that there are no conflicts of interest.

## Supporting information


Table S1

Table S2

Table S3

Table S4

## Data Availability

The data that support the findings of this study are available from the corresponding author upon reasonable request.
