# Supplementary material for: Influence of cyclin D1 splicing variants expression on breast cancer chemoresistance via CDK4/CyclinD1‐pRB‐E2F1 pathway
Source: J Cell Mol Med. 2023 Mar 13;27(7):991–1005. doi: 10.1111/jcmm.17716 (PMC10064037; doi:10.1111/jcmm.17716)
Supplement: Supplementary file 1 — Table S1 [file JCMM-27-991-s004.docx]

**TABLE S1** Primers Sequences for PCR and RT-PCR.

| **Primers Name** |  | **Sequences** | **Length** |
| --- | --- | --- | --- |
| CCND1a | Forward | 5′-TGGTGAACAAGCTCAA GTGGAACC-3′ | 259bp |
|  | Reverse | 5′-GTGAGGCGGTAGTAGGACAGGAAG-3′ |  |
| CCND1b | Forward | 5′-AACAGATCATCCGCAAACACGC-3′ | 224bp |
|  | Reverse | 5′-GCCTGGGACATCACCCTCACTT-3′ |  |
| β-actin | Forward | 5′-AGGATTCCTATGTGGGCGAC-3′ | 272bp |
|  | Reverse | 5′-ATAGCACAGCCTGGATAGCAA-3′ |  |
| CCND1-DNA | Forward | 5′-GTGAAGTTCATTTCCAATCCGC-3′ | 167bp |
|  | Reverse | 5′-GGGACATCACCCTCACTTAC-3′ |  |
