# Supplementary material for: Influence of cyclin D1 splicing variants expression on breast cancer chemoresistance via CDK4/CyclinD1‐pRB‐E2F1 pathway
Source: J Cell Mol Med. 2023 Mar 13;27(7):991–1005. doi: 10.1111/jcmm.17716 (PMC10064037; doi:10.1111/jcmm.17716)
Supplement: Supplementary file 2 — Table S2 [file JCMM-27-991-s001.docx]

**TABLE S2** Sensitivity (IC50) of MCF-7 and MCF-7/ADM to adriamycin.

| **Agent** | **IC50 (mean ± SD, µg/ml)** | | ***P*** |
| --- | --- | --- | --- |
|  | **MCF-7** | **MCF-7/ADM** |  |
| **Adriamycin** | 8.327±1.674 | 141.700±9.036 | 0.0001 |
