# Supplementary material for: Influence of cyclin D1 splicing variants expression on breast cancer chemoresistance via CDK4/CyclinD1‐pRB‐E2F1 pathway
Source: J Cell Mol Med. 2023 Mar 13;27(7):991–1005. doi: 10.1111/jcmm.17716 (PMC10064037; doi:10.1111/jcmm.17716)
Supplement: Supplementary file 3 — Table S3 [file JCMM-27-991-s002.docx]

**TABLE S3** The Hardy–Weinberg equilibrium of *CCND1* G870A polymorphism.

| **Group** | **Genotype** | | | ***P*** |
| --- | --- | --- | --- | --- |
|  | **GG** | **GA** | **AA** |  |
| **ChemoS** | 33 | 90 | 34 | 0.066 |
| **ChemoR** | 11 | 36 | 30 | 0.970 |
