# Supplementary material for: Influence of cyclin D1 splicing variants expression on breast cancer chemoresistance via CDK4/CyclinD1‐pRB‐E2F1 pathway
Source: J Cell Mol Med. 2023 Mar 13;27(7):991–1005. doi: 10.1111/jcmm.17716 (PMC10064037; doi:10.1111/jcmm.17716)
Supplement: Supplementary file 4 — Table S4 [file JCMM-27-991-s003.docx]

**TABLE S4** The association between the *CCND1* G870A polymorphism and BC chemoresistance risk in the subgroups.

|  | **Model** | **Allele/Genotype** | **Chemosensitive** | **Chemoresistant** | **OR (95%CI)** | ***P*** |
| --- | --- | --- | --- | --- | --- | --- |
| <48 | Heterozygote | GG | 16 (0.102) | 6 (0.078) |  |  |
|  |  | GA | 43 (0.274) | 13 (0.169) | 0.806 (0.262-2.483) | 0.707 |
|  | Homozygote | GG | 16 (0.102) | 6 (0.078) |  |  |
|  |  | AA | 21 (0.134) | 14 (0.182) | 1.778 (0.559-5.652) | 0.327 |
|  | Dominant | GG | 16 (0.102) | 6 (0.078) |  |  |
|  |  | GA+AA | 64 (0.408) | 27 (0.351) | 1.125 (0.397-3.184) | 0.824 |
|  | Recessive | AA | 21 (0.134) | 14 (0.182) |  |  |
|  |  | GA+GG | 59 (0.376) | 19 (0.247) | 0.483 (0.206-1.132) | 0.091 |
|  | Additive | GA | 43 (0.274) | 13 (0.169) |  |  |
|  |  | GG+AA | 37 (0.236) | 20 (0.260) | 1.788 (0.783-4.080) | 0.165 |
|  | Allele | G | 75 (0.469) | 25 (0.379) |  |  |
|  |  | A | 85 (0.531) | 41 (0.621) | 1.447 (0.805-2.601) | 0.216 |
| ≥48 | Heterozygote | GG | 17 (0.108) | 5 (0.065) |  |  |
|  |  | GA | 47 (0.299) | 23 (0.299) | 1.664 (0.546-5.073) | 0.368 |
|  | Homozygote | GG | 17 (0.108) | 5 (0.065) |  |  |
|  |  | AA | 13 (0.083) | 16 (0.208) | 4.185 (1.215-4.415) | **0.020** |
|  | Dominant | GG | 17 (0.108) | 5 (0.065) |  |  |
|  |  | GA+AA | 60 (0.382) | 39 (0.507) | 2.210 (0.754-6.479) | 0.142 |
|  | Recessive | AA | 13 (0.083) | 16 (0.208) |  |  |
|  |  | GA+GG | 64 (0.407) | 28 (0.364) | 0.355 (0.151-0.837) | **0.016** |
|  | Additive | GA | 47 (0.299) | 23 (0.299) |  |  |
|  |  | GG+AA | 30 (0.191) | 21 (0.273) | 1.430 (0.677-3.023) | 0.348 |
|  | Allele | G | 81 (0.526) | 33 (0.375) |  |  |
|  |  | A | 73 (0.474) | 55 (0.625) | 1.849 (1.083-3.158) | **0.024** |
| Postmenopausal | Heterozygote | GG | 9 (0.057) | 4 (0.052) |  |  |
|  |  | GA | 25 (0.159) | 14 (0.182) | 1.260 (0.328-4.847) | 1.000* |
|  | Homozygote | GG | 9 (0.057) | 4 (0.052) |  |  |
|  |  | AA | 9 (0.057) | 7 (0.091) | 1.750 (0.376-8.140) | 0.702* |
|  | Dominant | GG | 9 (0.057) | 4 (0.052) |  |  |
|  |  | GA+AA | 34 (0.216) | 21 (0.273) | 1.390 (0.380-5.086) | 0.754 |
|  | Recessive | AA | 9 (0.057) | 7 (0.091) |  |  |
|  |  | GA+GG | 34 (0.216) | 18 (0.234) | 0.681 (0.217-2.131) | 0.508 |
|  | Additive | GA | 25 (0.159) | 14 (0.182) |  |  |
|  |  | GG+AA | 18 (0.114) | 11 (0.143) | 1.091 (0.403-2.953) | 0.863 |
|  | Allele | G | 43 (0.500) | 22 (0.440) |  |  |
|  |  | A | 43 (0.500) | 28 (0.560) | 1.273 (0.632-2.564) | 0.499 |
| Premenopausal | Heterozygote | GG | 24 (0.153) | 7 (0.091) |  |  |
|  |  | GA | 65 (0.414) | 22 (0.286) | 1.160 (0.440-3.064) | 0.764 |
|  | Homozygote | GG | 24 (0.153) | 7 (0.091) |  |  |
|  |  | AA | 25 (0.159) | 23 (0.299) | 3.154 (1.144-8.701) | **0.023** |
|  | Dominant | GG | 24 (0.153) | 7 (0.091) |  |  |
|  |  | GA+AA | 90 (0.573) | 45 (0.585) | 1.714 (0.687-4.279) | 0.244 |
|  | Recessive | AA | 25 (0.159) | 23 (0.299) |  |  |
|  |  | GA+GG | 89 (0.567) | 29 (0.377) | 0.354 (0.175-0.716) | **0.003** |
|  | Additive | GA | 65 (0.414) | 22 (0.286) |  |  |
|  |  | GG+AA | 49 (0.312) | 30 (0.390) | 1.809 (0.932-3.512) | 0.078 |
|  | Allele | G | 113 (0.496) | 36 (0.346) |  |  |
|  |  | A | 115 (0.504) | 68 (0.654) | 1.856 (1.148-3.000) | **0.011** |
| size≤2 | Heterozygote | GG | 9 (0.057) | 5 (0.065) |  |  |
|  |  | GA | 26 (0.166) | 8 (0.104) | 0.554 (0.144-2.137) | 0.388 |
|  | Homozygote | GG | 9 (0.057) | 5 (0.065) |  |  |
|  |  | AA | 4 (0.025) | 12 (0.156) | 5.400 (1.120-6.044) | 0.063* |
|  | Dominant | GG | 9 (0.057) | 5 (0.065) |  |  |
|  |  | GA+AA | 30 (0.191) | 20 (0.260) | 1.200 (0.350-4.109) | 1.000 |
|  | Recessive | AA | 4 (0.025) | 12 (0.156) |  |  |
|  |  | GA+GG | 35 (0.223) | 13 (0.169) | 0.124 (0.034-0.454) | **0.001*** |
|  | Additive | GA | 26 (0.166) | 8 (0.104) |  |  |
|  |  | GG+AA | 13 (0.082) | 17 (0.221) | 4.250 (1.455-2.416) | **0.007** |
|  | Allele | G | 44 (0.564) | 18 (0.360) |  |  |
|  |  | A | 34 (0.436) | 32 (0.640) | 2.301 (1.108-4.777) | **0.024** |
| 2<size≤5 | Heterozygote | GG | 14 (0.089) | 5 (0.065) |  |  |
|  |  | GA | 35 (0.223) | 15 (0.195) | 1.200 (0.366-3.932) | 0.763 |
|  | Homozygote | GG | 14 (0.089) | 5 (0.065) |  |  |
|  |  | AA | 17 (0.108) | 8 (0.104) | 1.318 (0.351-4.945) | 0.682 |
|  | Dominant | GG | 14 (0.089) | 5 (0.065) |  |  |
|  |  | GA+AA | 52 (0.331) | 23 (0.299) | 1.238 (0.399-3.845) | 0.711 |
|  | Recessive | AA | 17 (0.108) | 8 (0.104) |  |  |
|  |  | GA+GG | 49 (0.312) | 20 (0.260) | 0.867 (0.323-2.330) | 0.778 |
|  | Additive | GA | 35 (0.223) | 15 (0.195) |  |  |
|  |  | GG+AA | 31 (0.197) | 13 (0.169) | 0.978 (0.403-2.374) | 0.962 |
|  | Allele | G | 63 (0.477) | 25 (0.446) |  |  |
|  |  | A | 69 (0.523) | 31 (0.554) | 1.132 (0.604-2.121) | 0.698 |
| Size>5 | Heterozygote | GG | 10 (0.064) | 1 (0.013) |  |  |
|  |  | GA | 29 (0.185) | 13 (0.169) | 4.483 (0.518-8.762) | 0.251* |
|  | Homozygote | GG | 10 (0.064) | 1 (0.013) |  |  |
|  |  | AA | 13 (0.083) | 10 (0.130) | 7.692 (0.840-0.457) | 0.060* |
|  | Dominant | GG | 10 (0.064) | 1 (0.013) |  |  |
|  |  | GA+AA | 42 (0.268) | 23 (0.299) | 5.476 (0.659-5.512) | 0.157* |
|  | Recessive | AA | 13 (0.083) | 11 (0.130) |  |  |
|  |  | GA+GG | 39 (0.249) | 14 (0.182) | 0.424 (0.155-1.163) | 0.092 |
|  | Additive | GA | 29 (0.185) | 13 (0.169) |  |  |
|  |  | GG+AA | 23 (0.147) | 11 (0.143) | 1.067 (0.404-2.819) | 0.896 |
|  | Allele | G | 49 (0.471) | 15 (0.300) |  |  |
|  |  | A | 55 (0.529) | 35 (0.700) | 2.079 (1.015-4.258) | **0.044** |

OR, Odds ratio; CI, Confidence interval.

*: Pass Fisher's exact test calculation.
